# Can non-pharmacological interventions change levels of neurofilament light in older adults at risk of dementia? A secondary analysis of the SCD-Well randomized clinical trial

**DOI:** 10.1016/j.tjpad.2025.100299

**Published:** 2025-07-18

**Authors:** Lehané Masebo, Tim Whitfield, Harriet Demnitz-King, Amanda Heslegrave, Géraldine Poisnel, Antoine Lutz, Eric Frison, Miranka Wirth, Abdul Hye, Frank Jessen, Nicholas J. Ashton, Henrik Zetterberg, Natalie L. Marchant

**Affiliations:** a Division of Psychiatry, University College London, London, United Kingdom; b Centre for Psychiatry and Mental Health, Wolfson Institute of Population Health, Queen Mary University, London, UK; c Dementia Research Institute, UCL, London, UK; d Department of Neurodegenerative Disease, Institute of Neurology, London, UK; e Normandie University, UNICAEN, INSERM, U1237, PhIND "Physiopathology and Imaging of Neurological Disorders", NeuroPresage Team, Institut Blood and Brain @ Caen-Normandie, Cyceron, 14000 Caen, France; f Lyon Neuroscience Research Center Inserm U1028, CNRS UMR5292, Lyon 1 University, Lyon, France; g Univ. Bordeaux, INSERM, Institut Bergonié, CHU Bordeaux, CIC1401-EC, EUCLID/F-CRIN clinical trials platform, F-33000 Bordeaux, France; h German Center for Neurodegenerative Diseases (DZNE), Dresden, Germany; i Department of Old Age Psychiatry, King's College London Institute of Psychiatry Psychology and Neuroscience, London, UK; j Department of Psychiatry, Medical Faculty, University of Cologne, Cologne, Germany; k Excellence Cluster on Cellular Stress Responses in Aging-Associated Diseases (CECAD), University of Cologne, Germany; l German Center for Neurodegenerative Diseases (DZNE), Bonn, Germany; m Department of Psychiatry and Neurochemistry, Institute of Neuroscience and Physiology, the Sahlgrenska Academy at the University of Gothenburg, Mölndal, Sweden; n Centre for Age-Related Medicine, Stavanger University Hospital, Stavanger, Norway; o NIHR Biomedical Research Centre for Mental Health and Biomedical Research Unit for Dementia, South London and Maudsley NHS Foundation, London, UK; p Hong Kong Center for Neurodegenerative Diseases, Clear Water Bay, Hong Kong, China; q Wisconsin Alzheimer’s Disease Research Center, University of Wisconsin School of Medicine and Public Health, University of Wisconsin-Madison, Madison, WI, USA

**Keywords:** Blood-based biomarkers, Fluid biomarkers, Blood, Psychological intervention, Behavior change

## Abstract

**Background:**

Older adults with subjective cognitive decline (SCD) and/or elevated neurofilament light (NfL), a neurodegeneration biomarker, are at increased risk of dementia. Non-pharmacological interventions offer a promising strategy for reducing dementia risk, yet none have utilized NfL as a marker of response in dementia prevention trials.

**Objective:**

To investigate the effects of two non-pharmacological interventions on NfL in older adults with SCD.

**Design:**

SCD-Well was an 8-week observer-blinded, randomized, clinical trial with 6-month follow-up, and was a part of the Horizon 2020 European Union-funded "Medit-Ageing" project. Data were analyzed from June 2022 to August 2024.

**Setting:**

Memory clinics at four sites in France, Germany, Spain, and UK.

**Participants:**

Participants were enrolled from March 2017 to January 2018 after fulfilling SCD research criteria and performing within the normal range on cognitive testing. Of the 147 participants enrolled, 140 were included in this secondary analysis (7 did not consent to venipuncture).

**Interventions:**

Participants were randomly allocated to the Caring Mindfulness-Based Approach for Seniors (CMBAS) intervention or a structurally matched Health Self-Management Program (HSMP).

**Measurements:**

Plasma NfL was measured at baseline (V1), post-intervention (V2), and 6-month follow-up (V3), using Single molecule array technology, and log-transformed for analyses.

**Results:**

137 older adults with SCD provided NfL data (mean [SD] age: 72.7 [6.8] years; 62.0 % female; CMBAS, *n* = 70; HSMP, *n* = 67). NfL data were available at V1 (*n* = 136), V2 (*n* = 119) and V3 (*n* = 115). The visit-by-arm interaction was not statistically significant, and no significant changes in NfL were observed within the CMBAS or HSMP arms from V1 to V2. However, within the HSMP arm, NfL levels reduced from V1 to V3 (-0.10, 95 % confidence interval [-0.18 to -0.02]). Modified intention-to-treat analyses, which included 140 participants, supported these findings, and additionally recorded significant reductions in the HSMP arm from V1 to V2 (*n* = 140, -0.07 [-0.14 to -0.00]).

**Conclusions:**

In this study, NfL levels were reduced at 6-month follow-up after a health self-management program. Future interventions with longer duration, extended follow-up and clinical endpoints will help clarify whether NfL reductions are sustained over extended timeframes and translate to lower dementia incidence.

**Trial registration:**

ClinicalTrials.gov Identifier: (NCT03005652).

## 1 Introduction

Dementia is a clinical syndrome defined by severe cognitive impairment, and subjective cognitive decline (SCD) is associated with an increased risk of incident dementia [1]. SCD is characterized by a perception of worsening cognition despite unimpaired objective cognition [2]. Most neuropathological processes start years before dementia onset, and evidence supporting the role of modifiable risk factors in dementia risk is growing. Targeting modifiable risk factors in high-risk groups such as those with SCD may help prevent dementia [3].

Neurofilament light chain (NfL) is a cytoskeletal protein that is released following axonal damage or neuronal degeneration. NfL measured in cerebrospinal fluid or, more recently, blood [4] is increasingly harnessed as a biomarker of neurological disease. NfL levels differentiate SCD from mild cognitive impairment (MCI) and neurodegenerative diseases [5,6], and predict objective cognitive decline in individuals with SCD [7]. Indeed, average NfL plasma levels increase 3.4 times faster in individuals who go on to develop Alzheimer’s disease (AD, the leading cause of dementia) compared to those who remain dementia-free over 14 years [8]. Reducing NfL levels could therefore reduce dementia risk; however, no trial has yet employed NfL as a surrogate endpoint in individuals with SCD.

Recent research suggests that psycho-affective factors may be associated with dementia risk, including perceived stress [9], anxiety [10], neuroticism [11], repetitive negative thinking [12], and sleep health [13]. Meditation practices (encompassing both mindfulness- and compassion-focused approaches) positively impact psycho-affective health [14]. Mindfulness-based interventions (MBIs) have been evaluated particularly intensively, with evidence syntheses reporting salutary effects on inflammatory [15], psychiatric [16], sleep [17], and cognitive [18] outcomes, as well as structural brain changes [19]. These findings motivated the hypothesis that MBIs could reduce dementia risk [20].

Health self-management programs (HSMPs) have often been used as comparator conditions in MBI trials [21], and have shown efficacy on health-related outcomes in their own right [22,23]. Topics include management of sleep, stress, physical activity, healthy eating and future planning [24]. Given some of these factors have been linked to dementia risk [3] and/or NfL [25,26], a HSMP could benefit dementia-related outcomes.

This study reports NfL outcome data from the SCD-Well trial, which examined the effects of an 8-week mindfulness- and compassion-based intervention (Caring Mindfulness-Based Approach for Seniors; CMBAS) versus a matched HSMP in older adults with SCD [24]. We previously reported that participants in both trial arms had reduced anxiety symptoms (primary outcome) [27] and improved cognition [28] at 6-month follow-up; the HSMP group also showed an increase in self-reported physical activity at follow-up [29]. Based on these earlier findings, we hypothesized that participants in both trial arms would show significant reductions in NfL levels from baseline to 6-month follow-up.

## 2 Methods

### 2.1 Study design

SCD-Well was a multicenter, observer-blinded, randomized, controlled trial [24] with two parallel 8-week arms (CMBAS or HSMP) and a 6-month follow-up (ClinicalTrials.gov Identifier: NCT03005652). The trial took place in memory clinics at four European sites (Cologne, Germany; London, UK; Barcelona, Spain; and Lyon, France). SCD-Well was sponsored by the French National Institute of Health and Medical Research (INSERM) and ethical approval was obtained at each site.

### 2.2 Participants

Participants were aged ≥ 60y, fulfilling SCD research criteria [2], performed within the normal range on cognitive testing (to exclude MCI), visited a memory clinic due to cognitive concerns, and had capacity to provide informed consent. They had no major neurological/psychiatric disorder, history of cerebral disease, visual/auditory impairment, current medication that might interfere with cognition, or regular engagement with meditation. Full eligibility criteria are detailed in the study protocol [24].

### 2.3 Procedures

Participants were recruited in two waves at each site. After providing written consent, including optional consent for a) blood sampling and b) genetic analysis, participants were invited to the baseline visit (V1) for venipuncture and behavioral assessments. These procedures were repeated at a post-intervention visit (V2) and at a follow-up visit (V3) six months after randomization.

### 2.4 Randomization and masking

After 14–25 participants in each site had completed V1, these participants were randomized – 1:1 allocation, permuted blocks of size 4 and 6, stratified by site – on the same day via a centralized procedure implemented by the EUCLID clinical trials platform. The research team (except trial managers) were blind to participants’ allocations.

### 2.5 Interventions

#### 2.5.1 Caring mindfulness-based approach for seniors (CMBAS)

The CMBAS followed the format of a mindfulness-based stress reduction program, consisting of a pre-class interview, eight weekly 2 h group-based sessions, and a half-day meditation workshop. CMBAS participants were also taught compassion meditation practices focusing on cultivating wholesome attitudes toward oneself and others. It was specifically tailored to older adults’ needs, building on an earlier study which developed an MBI for seniors [30]. Participants were asked to engage in home practice for approximately 1h/day for 6d/week, consisting of guided meditations and informal practices designed to embed mindfulness skills in daily life.

#### 2.5.2 Health self-management program (HSMP)

The HSMP followed the same format and structure as CMBAS, and was matched in administration, dosage, intensity, and duration. The HSMP controlled for nonspecific interventional factors (e.g., social interaction, professional facilitator, physical activity) and treatment expectancies. The HSMP was based on a published manual aimed at helping people live well with long-term health conditions based on a self-efficacy model [31]. The program was previously adapted and validated for SCD participants [21]. The HSMP included the following topics: management of sleep, stress, exercise, medicines and memory; communication; healthy eating; and future planning. Participants were asked to create and implement weekly ‘action plans’ to promote engagement in activities based on their preferences.

### 2.6 Measures

#### 2.6.1 Plasma biomarkers

Peripheral EDTA blood samples were taken via venipuncture, centrifuged (ideally within 2 h of collection), and frozen in 0.5 ml fluidx aliquots at −80 °C. Plasma NfL, Aβ_40_, Aβ_42_, and phosphorylated-tau-181 (p-tau-181) concentrations were measured by Single molecule array (Simoa) on an HD-X analyzer (Quanterix), using commercially-available kits according to the manufacturer’s instructions. Concentrations of NfL and p-tau-181 were measured at all visits; Aβ_40_ and Aβ_42_ were only measured at baseline. Briefly, samples were thawed at 21 °C, vortexed, and centrifuged at 13,000 RCF for five minutes at 21 °C. On-board the instrument, samples were diluted 1:4 with sample diluent and bound to paramagnetic beads coated with capture antibodies specific for each analyte. Analyte-bound beads were then incubated with biotinylated detection antibodies in turn conjugated to streptavidin-β-galactosidase complex acting as a fluorescent tag. Subsequent hydrolysis reaction with a resorufin β-D-galactopyranoside substrate produces a fluorescent signal proportional to the concentration of the analyte. Sample concentrations were extrapolated from a standard curve, fitted using a 4-parameter logistic algorithm. Plasma biomarker concentrations are reported in picograms per milliliter (pg/ml).

#### 2.6.2 Additional measures

Physical activity was measured using The Physical Activity Scale for the Elderly (PASE; global score). Global cognition was measured using The Mattis Dementia Rating Scale-2 (DRS-2; global score). Trait anxiety symptoms were assessed using the State-Trait Anxiety Inventory trait subscale (trait-STAI). Participants’ medical history was ascertained via a self-report questionnaire, including presence/absence of history of kidney disease. Body mass index (BMI; kg/m^2^) was calculated from participants’ height and weight.

### 2.7 Statistical analyses

Sample size calculations were based on the expected between-arm effect size for the pre- to post-intervention change in trait-STAI (primary outcome) with 80 % power and two-sided type 1 error of 5 % [24]. This resulted in a minimum sample of 128 participants (64 per group), which the trial exceeded. Descriptive statistics were used to summarize demographic and baseline variables for each arm (see Table 1). Exploratory analyses were conducted to characterize the relationships between (untransformed) plasma NfL values and other key variables (i.e., age, sex, education, PASE, DRS-2, trait-STAI, kidney disease, BMI, p-tau-181 and Aβ_42_/Aβ_40_) at baseline. Baseline NfL data were right-skewed therefore non-parametric correlations and *t*-tests were used for these analyses.

**Table 1:** SCD-Well sample baseline characteristics (participants with ≥ 1 NfL value available).

	Trial arm	
Variable	HSMP (*n* = 67)	CMBAS (*n* = 70)
Site		
Barcelona	20 (30 %)	20 (28 %)
Cologne	19 (28 %)	18 (26 %)
London	14 (21 %)	14 (20 %)
Lyon	14 (21 %)	18 (26 %)
Sex (female)	41 (61 %)	44 (63 %)
Ethnicity		
Asian	1 (2 %)	1 (1 %)
White	66 (98 %)	66 (95 %)
Mixed	0 (0 %)	2 (3 %)
Other	0 (0 %)	1 (1 %)
Age (years)	73.3 (5.9)	72.1 (7.6)
Education (years)	13.3 (3.5)	13.9 (3.8)
NfL (pg/ml)		
Untransformed	26.7 (15.3)	24.1 (12.7)
Log-transformed	3.1 (0.7)	3.1 (0.5)
Aβ_42_/Aβ_40_ (pg/ml)	0.06 (0.04)	0.06 (0.01)
P-tau-181 (pg/ml)	1.8 (1.1)	1.7 (0.9)
PASE (global score; range 0–400)	117.5 (65.2)	132.0 (75.5)
DRS-2 (global score; range 0–144)	139.3 (3.5)	140.2 (3.8)
Trait-STAI (range 20–80)	38.6 (9.4)	41.0 (10.2)
BMI (kg/m^2^)	26.0 (4.3)	26.1 (3.7)
Renal insufficiency (yes)	1 (1 %)	6 (9 %)
*APOE* (ε4 carrier)	19 (31 %)	21 (30 %)

Data shown as *n* ( %) or mean (SD). BMI = body mass index. CMBAS = caring mindfulness-based approach for seniors. DRS-2 = Mattis dementia rating scale-2. HSMP = health self-management program. NfL = neurofilament light. PASE = physical activity scale for the elderly. Trait-STAI = state-trait anxiety inventory (trait subscale). *APOE* = apolipoprotein E gene. Aβ = amyloid-beta peptide. P-tau-181 = tau phosphorylated at threonine 181. In the HSMP arm, there were missing data in baseline NfL (*n* = 1), P-tau-181 (*n* = 1), PASE (*n* = 3) and *APOE* (*n* = 5). In the CMBAS arm, there were missing data in P-tau-181 (*n* = 1), PASE (*n* = 2), DRS-2 (*n* = 1), trait-STAI (*n* = 1) and *APOE* (*n* = 1).

Linear mixed models were fit via restricted maximum likelihood to estimate the between- and within-arm effect of interventions on plasma NfL. Residuals of initial models were non-normally distributed, thus plasma NfL data were natural logarithm (log)-transformed for all linear mixed model analyses reported here, resulting in residuals better approximating a normal distribution. The difference between two logged numbers (i.e., change in log plasma NfL) can be interpreted as a percentage difference on the original (i.e., untransformed) scale. We therefore report both change in log NfL, as well as percentage change for untransformed NfL. For each analysis (see below), an unadjusted model, as well as three adjusted models were fit. The unadjusted model (Model 1) included random participant intercepts, as well as the fixed effects of visit (coded as ‘baseline’, ‘post-intervention’ and ‘follow-up’), site, trial arm, and the visit-by-trial arm interaction. Model 2 further adjusted Model 1 for the fixed effects of baseline age, sex, and education level. Following previous work, a third model (Model 3) additionally adjusted for kidney disease [32] as well as baseline BMI [25] and PASE [26,33]. A final model (Model 4) further adjusted for *APOE* genotype (coded as ε4 carrier/non-carrier) and baseline plasma biomarker (i.e., p-tau-181 and Aβ_42_/Aβ_40_) concentrations. Continuous covariates were mean-centered. Both observed data and modified intention-to-treat (mITT) analyses were performed.

#### 2.7.1 Observed data analysis

For observed data analyses, each linear mixed model included all available NfL data from participants with complete covariates; missing data were not replaced. In sensitivity analyses, motivated by previous research adjudging four MBI sessions to be an adequate minimal dose [34], models were re-estimated using only the subset of participants who attended ≥ 4 intervention sessions.

#### 2.7.2 Modified intention-to-treat (mITT) analysis

There were missing data for NfL and some covariates. To perform mITT analyses, missing data were replaced using multiple imputation by joint modeling. The mITT analyses only included randomized individuals who consented to venipuncture. Data were imputed separately for each trial arm to preserve any potential visit-by-arm interaction. A total of 20 datasets were ‘completed’ via multiple imputation, following which models were re-estimated for each dataset separately, and the results pooled according to Rubin’s rules.

Analyses were conducted in R 4.3.1 under RStudio 2024.04.2 + 764. Linear mixed models were fit using the *lme4* 1.1–35.5 package; *p*-values were obtained via *lmerTest* 3.1–3. The *anova.lmerModLmerTest* function was used to perform an analysis of variance (ANOVA) for the 3-level visit-by-arm interaction (deriving an omnibus *p*-value). The *emmeans* 1.10.3 package was used to compute within-arm change in log NfL for each arm separately (comprising two contrasts: baseline to post-intervention, and baseline to follow-up). Multiple imputation by joint modeling was performed using the *mitml* 0.4–5 package; imputations took the multilevel (i.e., nesting of NfL observations within individuals) structure of the data into account. For all analyses, *p*-values were deemed statistically significant at < 0.05.

## 3 Results

**Fig. 1:**
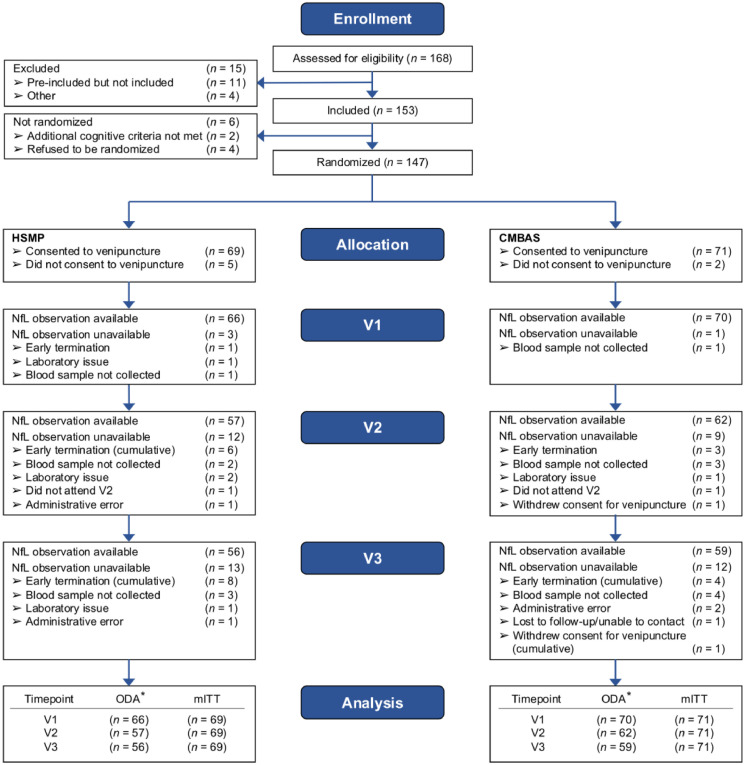
CONSORT flow of enrollment and randomization to HSMP and CMBAS Superscript: *These *n*s refer to the total number of NfL values available for observed data analysis; the actual *n*s used to estimate each model depended on the included covariates (see main text for details). CMBAS = caring mindfulness-based approach for seniors. HSMP = health self-management program. NfL = neurofilament light. V1 = baseline visit. V2 = post-intervention visit. V3 = follow-up visit. ODA = observed data analysis. mITT = modified intention-to-treat analysis.

Enrollment was from March 23rd, 2017 to January 25th, 2018. A total of 147 participants with SCD (mean age 72.7 ± 6.9 years; 64 % female) were randomized. Of these, 140 (95 %) individuals consented to venipuncture (for non-genetic analyses), and 136 also consented to genetic (*APOE*) analysis. The mITT analyses thus included a maximum of 140 individuals; observed data analyses included fewer individuals. Table 1 shows the sample baseline characteristics and Fig. 1 provides the CONSORT diagram.

### 3.1 Associations between untransformed baseline nfl and key variables

At baseline, median [interquartile range; IQR] NfL levels (pg/ml) did not differ by sex (female = 21.7 [16.9–30.6], male = 22.6 [16.5–29.8]; *p* = 0.88) or kidney disease (present = 22.5 [15.5–26.9], absent = 22.2 [16.9–30.5]; *p* = 0.70). NfL levels were positively correlated with age (ρ = 0.48; *p* < 0.0001) and p-tau-181 (ρ = 0.51; *p* < 0.0001), and negatively correlated with Aβ_42_/Aβ_40_ (ρ = −0.38; *p* < 0.0001), BMI (ρ = −0.22; *p* = 0.010) and PASE (ρ = −0.21; *p* = 0.018). NfL was not associated with education (ρ = −0.02; *p* = 0.77), DRS-2 (ρ = −0.15; *p* = 0.088), or trait-STAI (ρ = −0.06; *p* = 0.50).

### 3.2 Intervention engagement

There was no significant difference between arms for the mean number of intervention sessions attended (CMBAS = 6.7 ± 2.9; HSMP = 7.1 ± 2.2; *p* = 0.62), the proportion of participants who attended ≥ 4 intervention sessions: CMBAS (*n* = 56; 80 %); HSMP (*n* = 59; 89 %; *p* = 0.20) or who reported continued engagement with intervention-related activities between V2 and V3: CMBAS (*n* = 37; 57 %); HSMP (*n* = 33; 54 %; *p* = 0.89).

### 3.3 Change in log plasma NfL between and within trial arms

#### 3.3.1 Observed data analysis

Appendix Table S1 provides the observed NfL values (untransformed and log-transformed) for each group at each visit. Participants with complete NfL data had significantly higher education than those with incomplete data but did not differ on any other baseline characteristics (see Appendix Table S2). The results for estimated change in log NfL between and within arms did not substantively differ across the four linear mixed models (i.e., Models 1–4; Table 2); here we report the results for Model 4 only. The ANOVA for the visit-by-arm interaction was not significant, indicating that change in log NfL did not differ between interventions (F(2, 211) = 0.87; *p* = 0.42).

**Table 2:** Linear mixed model estimates of within- and between-arm changes in log_e_ plasma NfL (observed data analysis).

		Estimate [95 % CI]			
		Model 1	Model 2	Model 3	Model 4
**Within-arm estimated change**					
HSMP	V2 – V1	−0.07 [−0.14 to 0.00]	−0.07 [−0.14 to 0.00]	−0.07 [−0.14 to 0.00]	−0.06 [−0.14 to 0.01]
	V3 – V1	−0.11 [−0.18 to −0.04]	−0.11 [−0.18 to −0.04]	−0.10 [−0.18 to −0.03]	−0.10 [−0.18 to −0.02]
CMBAS	V2 – V1	−0.04 [−0.11 to 0.02]	−0.04 [−0.11 to 0.02]	−0.05 [−0.11 to 0.02]	−0.05 [−0.12 to 0.02]
	V3 – V1	−0.02 [−0.09 to 0.04]	−0.02 [−0.09 to 0.04]	−0.03 [−0.10 to 0.04]	−0.03 [−0.10 to 0.04]
**Between-arm estimated change**					
HSMP – CMBAS	V2 – V1	−0.02 [−0.12 to 0.07] *p* = 0.64	−0.02 [−0.12 to 0.07] *p* = 0.63	−0.02 [−0.12 to 0.08] *p* = 0.65	−0.02 [−0.12 to 0.09] *p* = 0.77
	V3 – V1	−0.08 [−0.18 to 0.02] *p* = 0.11	−0.08 [−0.18 to 0.02] *p* = 0.10	−0.08 [−0.18 to 0.02] *p* = 0.13	−0.07 [−0.17 to 0.04] *p* = 0.21
**Data included in models**					
Participants		137	137	132*	124†
Observations		370	370	360	336

The unadjusted model (Model 1) included random participant intercepts (all other covariates were fixed effects), as well as trial site, visit, trial arm, and the visit × arm interaction. Model 2 adjusted Model 1 for age at baseline, sex, and education level. Model 3 adjusted Model 2 for renal insufficiency, as well as baseline BMI and PASE. Model 4 adjusted Model 3 for *APOE*. Continuous covariates were mean-centered. CI = confidence interval. CMBAS = caring mindfulness-based approach for seniors. HSMP = health self-management program. NfL = neurofilament light. PASE = physical activity scale for the elderly. V1 = baseline visit. V2 = post-intervention visit. V3 = follow-up visit. *APOE* = apolipoprotein E gene. P-tau-181 = tau phosphorylated at threonine 181.

⁎ Model 3 included 5 fewer participants versus Models 1–2 due to missingness in the PASE covariate.

† Model 4 included 8 fewer participants versus Model 3 due to missingness in the *APOE* (*n* = 6) and P-tau-181 (*n* = 2) covariates.

**Fig. 2:**
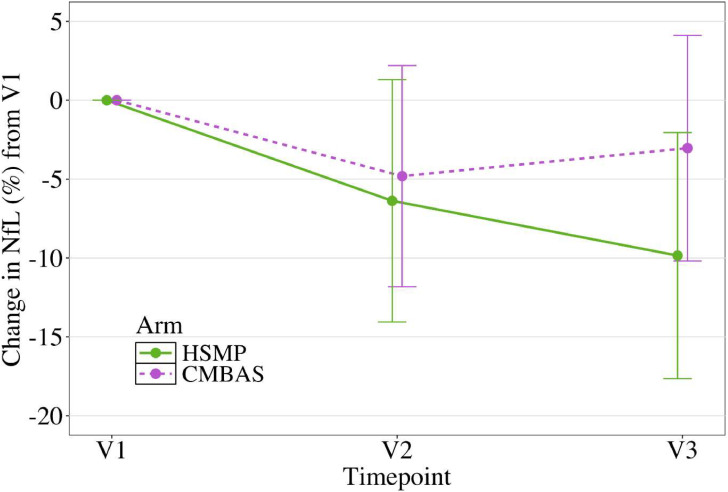
Percent change in NfL from baseline (V1) to post-intervention (V2) and follow-up (V3) for each intervention condition Caring mindfulness-based approach for seniors (CMBAS) is drawn in purple, Health self-management program (HSMP) is drawn in green. Standard error bars are shown.

Within the CMBAS arm, there was no significant change in log NfL from V1 to V2 (estimated change [95 % confidence interval]: −0.05 [−0.12 to 0.02]), or V1 to V3 (−0.03 [−0.10 to 0.04]). Within the HSMP arm, there was no significant change in log NfL from V1 to V2 (−0.06 [−0.14 to 0.01]). However, from V1 to V3 there was a significant reduction in log NfL (−0.10 [−0.18 to −0.02]). Expressed as percentage change on the non-logged NfL scale, from V1 to V2, there was a 4.8 % reduction in the CMBAS arm and a 6.4 % reduction in NfL in the HSMP arm. From V1 to V3, there was a 3.0 % reduction in the CMBAS arm and a 9.8 % reduction in NfL in the HSMP arm (Fig. 2). These results were substantively unchanged in sensitivity analyses (see Appendix Table S3).

#### 3.3.2 Modified intention-to-treat analysis

In all four mITT models, for which missing data in the outcome and covariates were replaced via multiple imputation, the reduction in log NfL from V1 to V2 in the HSMP arm became significant (−0.07 [−0.14 to −0.00]) (see Appendix Table S4). All other mITT results were substantively unchanged from observed data analyses.

## 4 Discussion

In this RCT of two 8-week interventions with longitudinal follow-up in older adults with SCD, we found no difference between interventions in change in NfL, a marker of neurodegeneration. We did, however find a significant reduction in NfL in the HSMP intervention from baseline to 6-month follow-up, which was not observed in the CMBAS intervention. Additional analyses confirmed these findings.

While both arms showed a downwards trajectory of NfL levels, the HSMP intervention marginally reduced NfL post-intervention and significantly reduced NfL at follow-up. We interpret this NfL decrease as a sign that the intervention may have slowed neurodegeneration. While we are unable to discern the underlying mechanism driving this change, we discuss potential mechanisms on the basis of the intervention’s characteristics. The HSMP targets some of the potentially modifiable risk factors for dementia identified in the Lancet Commission [3], including physical inactivity, depression, and various cardiometabolic factors (i.e., high LDL cholesterol, diabetes, hypertension and obesity). The salutary NfL trajectories observed in the HSMP arm may have resulted from the amelioration of any one or several of these risk factors due to the multi-component nature of the intervention, potentially reducing dementia risk. However, it is important to acknowledge that the amount of time dedicated to each of these (and the degree to which they are addressed directly versus indirectly) within the HSMP varies. That is, an entire session focused on increasing physical activity levels, whereas managing depressive symptoms was addressed as a subtopic within the sleep session. The cardiometabolic factors were addressed within the healthy eating session (delivered as a half-day workshop). In an earlier publication, we reported that participants in HSMP had increased (versus CMBAS) self-reported physical activity levels at follow-up [29]. The reduction in NfL may, therefore, have reflected the observed increase in physical activity in the HSMP arm [29]. The putative effect of the HSMP on NfL could be mediated via neuroinflammatory mechanisms, given lifestyle factors can modulate innate immune responses and microglia-driven inflammation in aging brains [35]. Activated microglia can support neuronal recovery through release of growth factors and removal of damaged neurons/myelin debris. Microglia also act as a cellular mediator of synaptic loss [36]. The behaviors targeted by the HSMP may thus have stimulated microglial activity and activated a protective response, though further research is required to evaluate this hypothesis.

We hypothesized that there would be no difference in changes in NfL between the two interventions due to evidence that behaviors targeted in each arm could potentially reduce dementia risk and/or NfL [25,27,28,33]. The present findings partially support this hypothesis, showing no significant difference between HSMP and CMBAS. The lack of a passive comparator precludes direct examination of whether reductions in NfL were specifically due to intervention. To partially address this limitation we identified longitudinal observational studies which reported plasma NfL data for cognitively unimpaired older adults or individuals with SCD, and extrapolated these data to match SCD-Well’s follow-up period (6 months; Appendix Table S5). Of five studies identified, all observed an increase in NfL (average = 3.9 %). This increase contrasts with the currently-observed reduction of 6.4 % (post-intervention) and 9.5 % (follow-up) in the HSMP, and 4.6 % and 2.7 % in the CMBAS. While only indicative and not confirmatory, this supports the idea that the HSMP reduced or attenuated NfL aggregation.

This study has several strengths. Chiefly, it is the first trial to use NfL as a secondary outcome in older adults with SCD, and included a larger sample versus previous trials that measured NfL in other populations [33]. SCD-Well included manualized interventions matched across key factors and adapted for older adults with SCD, and included a 6-month follow-up [24]. It also used strict inclusion criteria to both homogenize the sample with SCD and select individuals at elevated dementia risk [2], while excluding MCI [24].

The study also has limitations. SCD-Well was designed around the primary outcome (anxiety) [27], which may have resulted in low statistical power to detect changes in NfL. This study did not include a passive comparator, so we could not rule out that NfL levels could have reduced without intervention; neither did it include additional neurodegeneration biomarkers (e.g., brain volumetric changes), which would enable a more comprehensive assessment of effects on neurodegeneration. Further, whilst we collected blood-based biomarkers, we were unable to ascertain the presence/absence of AD pathology, as relevant cut-offs are not yet available for the research assays used in this study. Given the non-trivial amounts of unavailable NfL data at post-intervention (15 %) and follow-up (18 %), data were multiply imputed for mITT analyses. Whilst the use of multiple imputation to handle missingness in RCT analyses has increased [37], it is important to acknowledge that inferences based on imputed datasets need to be interpreted with caution, as the technique relies on the untestable assumption that data are missing at random [38].

In conclusion, this is the first trial targeting older adults with SCD to use NfL as a surrogate endpoint. Participants who underwent the health self-management intervention showed a significant reduction in NfL. While the mechanism(s) driving this reduction are not yet known, these findings highlight the promise of personalized lifestyle interventions targeting a combination of factors linked to dementia risk. Future trials including longer interventions, passive comparators and extended follow-up periods are needed to understand whether reductions in NfL can be sustained and translate to clinically meaningful outcomes.

## Ethics approval and consent to participate

This study was approved by Ethics Committees and regulatory agencies at all centers: London, UK (Queen Square Research Ethics Committee: No 17/LO/0056 and Health Research Authority IRAS project ID: 213,008); Lyon, France (Comité de Protection des Personnes Sud-Est II Groupement Hospitalier Est: No. 2016–30–1 and Agence Nationale de Sécurité du Médicament et des Produits de Santé: IDRCB 2016-A01298–43); Cologne, Germany (Ethikkommission der Medizinischen Fakultät der Universität zu Köln: No. 17–059); and Barcelona, Spain (Comité Etico de Investigacion Clinica del Hospital Clinic de Barcelona: No. HCB/2017/0062). Written informed consent to take part in the trial was secured from all participants after the procedures had been fully explained to them and prior to trial participation. To be eligible for inclusion in any of the present analyses, participants had to consent to venipuncture (for the purposes of analyzing biomarkers). Moreover, to be eligible for inclusion in models including *APOE* as a covariate, participants had to consent to genetic analysis being performed (both consent ‘clauses’ were optional). The authors assert that all the procedures contributing to this work comply with the ethical standards of the relevant national and institutional committees on human experimentation and with the Helsinki Declaration of 1975, as revised in 2008.

## Data sharing

The data underlying this report are made available on request following a formal data sharing agreement and approval by the consortium and executive committee (https://silversantestudy.eu/2020/09/25/data-sharing). The Material can be mobilized, under the conditions and modalities defined in the Medit-Ageing Charter by any research team belonging to an Academic, for carrying out a scientific research project relating to the scientific theme of mental health and well-being in older people. The Material may also be mobilized by non-academic third parties, under conditions, in particular financial, which will be established by separate agreement between Inserm and by the said third party. Data sharing policies described in the Medit-Ageing charter are in compliance with our ethics approval and guidelines from our funding body.

## Funding

The SCD-Well RCT is part of the Medit-Ageing project funded through the European Union in Horizon 2020 program related to the call PHC22 Promoting Mental Well-Being in the Ageing Population and under grant agreement No. 667,696. HZ is a Wallenberg Scholar supported by grants from the Swedish Research Council (#2022–01,018 and #2019–02,397), the European Union’s Horizon Europe research and innovation program under grant agreement No 101,053,962, Swedish State Support for Clinical Research (#ALFGBG-71,320), the Alzheimer Drug Discovery Foundation (ADDF), USA (#201,809–2016,862), the AD Strategic Fund and the Alzheimer's Association (#ADSF-21–831,376-C, #ADSF-21–831,381-C, and #ADSF-21–831,377-C), the Bluefield Project, the Olav Thon Foundation, the Erling-Persson Family Foundation, Stiftelsen för Gamla Tjänarinnor, Hjärnfonden, Sweden (#FO2022–0270), the European Union’s Horizon 2020 research and innovation program under the Marie Skłodowska-Curie grant agreement No 860,197 (MIRIADE), the European Union Joint Program – Neurodegenerative Disease Research (JPND2021–00,694), the National Institute for Health and Care Research University College London Hospitals Biomedical Research Centre, and the UK Dementia Research Institute at UCL (UKDRI-1003). The funders had no role in the study design, data acquisition, data analysis, data interpretation, or writing of the report.

## Declaration of generative AI and AI-assisted technologies in the writing process

Generative AI and AI-assisted technologies were not used during the preparation of this manuscript.

## CRediT authorship contribution statement

**Lehané Masebo:** Writing – review & editing, Writing – original draft, Formal analysis. **Tim Whitfield:** Writing – review & editing, Writing – original draft, Formal analysis, Data curation. **Harriet Demnitz-King:** Writing – review & editing, Data curation. **Amanda Heslegrave:** Writing – review & editing, Methodology. **Géraldine Poisnel:** Writing – review & editing. **Antoine Lutz:** Writing – review & editing. **Eric Frison:** Writing – review & editing. **Miranka Wirth:** Writing – review & editing. **Abdul Hye:** Writing – review & editing, Conceptualization. **Frank Jessen:** Writing – review & editing. **Nicholas J. Ashton:** Writing – review & editing, Methodology, Conceptualization. **Henrik Zetterberg:** Writing – review & editing, Methodology. **Natalie L. Marchant:** Writing – review & editing, Conceptualization.

## Declaration of competing interest

The authors declare the following financial interests/personal relationships which may be considered as potential competing interests: Henrik Zetterberg reports a relationship with Brain Biomarker Solutions in Gothenburg AB that includes: board membership. Henrik Zetterberg reports a relationship with Abbvie, Acumen, Alector, Alzinova, ALZPath, Amylyx, Annexon, Apellis, Artery Therapeutics, AZTherapies, Cognito Therapeutics, CogRx, Denali, Eisai, Merry Life, Nervgen, Novo Nordisk, Optoceutics, Passage Bio, Pinteon Therapeutics, Prothena, Red Abbey Labs, reMYND, Roche, Samumed, Siemens Healthineers, Triplet Therapeutics, and Wave that includes: consulting or advisory. Henrik Zetterberg reports a relationship with Alzecure, Biogen, Cellectricon, Fujirebio, Lilly, Novo Nordisk, and Roche that includes: speaking and lecture fees. Nicholas J. Ashton reports a relationship with Alamar Bioscience, and Quanterix Corp that includes: consulting or advisory. Nicholas J. Ashton reports a relationship with Alamar Bioscience, Quanterix Corp, and Eli-Lilly that includes: speaking and lecture fees. Amanda Heslegrave reports a relationship with Quanterix Corp that includes: consulting or advisory. Henrik Zetterberg is chair of the Alzheimer’s Association Global Biomarker Standardization Consortium. If there are other authors, they declare that they have no known competing financial interests or personal relationships that could have appeared to influence the work reported in this paper.

## Appendix Supplementary materials

Image, application 1

Image, application 2
