# Supplementary material for: Can non-pharmacological interventions change levels of neurofilament light in older adults at risk of dementia? A secondary analysis of the SCD-Well randomized clinical trial
Source: J Prev Alzheimers Dis. 2025 Jul 18;12(8):100299. doi: 10.1016/j.tjpad.2025.100299 (PMC12413708; doi:10.1016/j.tjpad.2025.100299)
Supplement: Supplementary file 1 [file mmc1.docx]

**Appendix**

**Can non-pharmacological interventions change levels of neurofilament light in older adults at risk of dementia?
A secondary analysis of the SCD-Well randomised clinical trial**

Lehané Masebo MSc, Tim Whitfield PhD, Harriet Demnitz-King PhD, Amanda Heslegrave PhD, Géraldine Poisnel PhD, Antoine Lutz PhD, Eric Frison MD, PhD Miranka Wirth PhD , Abdul Hye PhD, Frank Jessen MD, Nicholas J. Ashton PhD, Henrik Zetterberg MD, PhD & Natalie L. Marchant PhD for The Medit-Ageing Research Group.

**Additional File –** Supplementary Information

**Table S1:** Observed NfL values (untransformed and log-transformed) for each group at each visit.

**Table S2:** Comparison of baseline characteristics between individuals with all NfL observations available and those with ≥ 1 NfL observations unavailable.

**Table S3:** Linear mixed model estimates of within- and between-arm changes in log_e_ plasma NfL (observed data sensitivity analysis) – Data analysis to see if the full effect of the interventions would affect the change in NfL across the three timepoints

**Table S4:** Linear mixed model estimates of within- and between-arm changes in log_e_ plasma NfL (modified intention-to-treat analysis) – Data analysis using multiple imputation to replace missing data in the outcome and covariates

**Table S5:** Observational studies of longitudinal plasma NfL levels in older adults without cognitive impairment

**Table S1.** **Untransformed and log_e_ plasma NfL (pg/ml) levels by trial arm**

|  |  | **HSMP** | | **CMBAS** | |
| --- | --- | --- | --- | --- | --- |
| **Outcome** | **Visit** | **Mean (SD)** | ***n*** | **Mean (SD)** | ***n*** |
| Untransformed NfL | V1 | 26·7 (15·3) | 66 | 24·1 (12·7) | 70 |
|  | V2 | 24·0 (9·1) | 57 | 23·4 (15·7) | 62 |
|  | V3 | 23·1 (10·5) | 56 | 26·1 (27·5) | 59 |
| Log_e_ NfL | V1 | 3·13 (0·66) | *n*s as above | 3·06 (0·50) | *n*s as above |
|  | V2 | 3·07 (0·63) |  | 3·00 (0·53) |  |
|  | V3 | 2·98 (0·79) |  | 3·05 (0·58) |  |

CMBAS = caring mindfulness-based approach for seniors. HSMP = health self-management programme. NfL **=** neurofilament light. n = refers to the total number of NfL values available for observed data analysis.

**Table S2.** **Comparison of baseline characteristics between individuals with all NfL observations available and those with ≥ 1 NfL observations unavailable**

| **Variable** | **All NfL**  **observations available**  **(*n* = 110)** | **One or more NfL observations unavailable**  **(*n* = 30)** | ***p*** |
| --- | --- | --- | --- |
| Site  Barcelona  Cologne  London  Lyon | 35 (31·8)  28 (25·5)  23 (20·9)  24 (21·8) | 5 (16·7)  9 (30·0)  5 (16·7)  11 (36·7) | 0·22 |
| Sex (female) | 66 (60·0) | 22 (73·3) | 0·26 |
| Ethnicity  Asian  White  Mixed  Other | 1 (1%)  107 (97%)  2 (2%)  0 (0%) | 1 (3%)  28 (94%)  0 (0%)  1 (3%) | 0·16 |
| Age (years) | 72·9 (6·7) | 72·5 (7·1) | 0·81 |
| Education (years) | 13·3 (3·7) | 14·9 (3·2) | **0·037** |
| NfL (pg/ml)  Untransformed  Log-transformed | 24·6 (12·4)  3·1 (0·6) | 28·8 (19·5)  3·2 (0·6) | 0·17 0·39 |
| Aβ_42_/Aβ_40_ (pg/ml) | 0.06 (0.03) | 0.06 (0.01) | 0·95 |
| P-tau-181 (pg/ml) | 1.8 (1.0) | 1.7 (1.0) | 0·53 |
| PASE (global score) | 124·6 (67·0) | 122·2 (84·4) | 0·88 |
| DRS-2 (global score) | 139·9 (3·4) | 139·4 (4·3) | 0·54 |
| Trait-STAI | 39·9 (9·9) | 39·0 (10·3) | 0·66 |
| BMI (kg/m^2^) | 26·1 (3·9) | 25·6 (4·7) | 0·56 |
| Renal insufficiency (yes) | 5 (4·5) | 3 (10·0) | 0·49 |
| *APOE* (ε4 carrier) | 32 (30·8) | 8 (28·6) | 1·00 |

Data shown as *n* (%) or mean (SD). Inferential statistics are derived from parametric *t*-tests (for continuous variables), or chi-square/Fisher’s exact tests (for categorical variables). All *p*-values are two-sided and **emboldened** for < 0·05. BMI = body mass index. CMBAS = caring mindfulness-based approach for seniors. DRS-2 = Mattis dementia rating scale-2. HSMP = health self-management programme. NfL = neurofilament light. PASE = physical activity scale for the elderly. Trait-STAI = state-trait anxiety inventory (trait subscale). *APOE* = apolipoprotein E gene. Aβ = amyloid-beta peptide. P-tau-181 = tau phosphorylated at threonine 181.

**Table S3: Linear mixed model estimates of within- and between-arm changes in log_e_ plasma NfL (observed data sensitivity analysis)**

|  |  | **Estimate [95% CI]** | | | |
| --- | --- | --- | --- | --- | --- |
|  |  | **Model 1** | **Model 2** | **Model 3** | **Model 4** |
| **Within-arm estimated change** | | | | | |
| HSMP | V2 – V1 | -0·07 [-0·14 to 0·01] | -0·07 [-0·14 to 0·00] | -0·07 [-0·14 to 0·01] | -0·06 [-0·14 to 0·02] |
|  | V3 – V1 | -0·10 [-0·18 to -0·03] | -0·10 [-0·18 to -0·03] | -0·10 [-0·18 to -0·03] | -0·09 [-0·18 to -0·01] |
| CMBAS | V2 – V1 | -0·04 [-0·12 to 0·03] | -0·04 [-0·12 to 0·03] | -0·04 [-0·12 to 0·03] | -0·04 [-0·12 to 0·03] |
|  | V3 – V1 | -0·04 [-0·12 to 0·03] | -0·05 [-0·12 to 0·03] | -0·05 [-0·12 to 0·03] | -0·05 [-0·13 to 0·03] |
| **Between-arm estimated change** | | | | | |
| HSMP – CMBAS | V2 – V1 | -0·02 [-0·13 to 0·08] *p* = 0·64 | -0·02 [-0·13 to 0·08] *p* = 0·64 | -0·02 [-0·13 to 0·08] *p* = 0·65 | -0·02 [-0·13 to 0·09] *p* = 0·76 |
|  | V3 – V1 | -0·06 [-0·16 to 0·04] *p* = 0·26 | -0·06 [-0·16 to 0·04] *p* = 0·26 | -0·06 [-0·16 to 0·05] *p* = 0·29 | -0·05 [-0·16 to 0·06] *p* = 0·41 |
| **Data included in models** | | | | | |
| Participants | | 115 | 115 | 113^a^ | 105^b^ |
| Observations | | 330 | 330 | 325 | 301 |

These models only included the participants who attended at least 4 intervention sessions. CI = confidence interval; CMBAS = caring mindfulness-based approach for seniors; HSMP = health self-management programme; NfL = neurofilament light; V1 = baseline visit; V2 = post-intervention visit; V3 = follow-up visit. *APOE* = apolipoprotein E gene. P-tau-181 = tau phosphorylated at threonine 181. ^a^Model 3 included 2 fewer participants versus Models 1–2 due to missingness in the PASE covariate. ^b^Model 4 included 8 fewer participants versus Model 3 due to missingness in the *APOE* (*n* = 6) and P-tau-181 (*n* = 2) covariates.

**Table S4: Linear mixed model estimates of within- and between-arm changes in log_e_ plasma NfL (modified intention-to-treat analysis)**

|  |  | **Estimate [95% CI]** | | | |
| --- | --- | --- | --- | --- | --- |
|  |  | **Model 1** | **Model 2** | **Model 3** | **Model 4** |
| **Within-arm estimated change** | | | | | |
| HSMP | V2 – V1 | -0·07 [-0·14 to -0·00] | -0·07 [-0·14 to -0·00] | -0·07 [-0·14 to -0·00] | -0·07 [-0·14 to -0·00] |
|  | V3 – V1 | -0·10 [-0·17 to -0·03] | -0·10 [-0·17 to -0·03] | -0·10 [-0·17 to -0·03] | -0·10 [-0·17 to -0·03] |
| CMBAS | V2 – V1 | -0·04 [-0·11 to 0·03] | -0·04 [-0·11 to 0·03] | -0·04 [-0·11 to 0·03] | -0·04 [-0·11 to 0·03] |
|  | V3 – V1 | -0·02 [-0·09 to 0·05] | -0·02 [-0·09 to 0·05] | -0·02 [-0·09 to 0·05] | -0·02 [-0·09 to 0·05] |
| **Between-arm estimated change** | | | | | |
| HSMP – CMBAS | V2 – V1 | -0·03 [-0·12 to 0·07] *p* = 0·56 | -0·03 [-0·12 to 0·07] *p* = 0·56 | -0·03 [-0·12 to 0·07] *p* = 0·56 | -0·03 [-0·12 to 0·07] *p* = 0·56 |
|  | V3 – V1 | -0·08 [-0·18 to 0·02] *p* = 0·11 | -0·08 [-0·18 to 0·02] *p* = 0·11 | -0·08 [-018 to 0·02] *p* = 0·11 | -0·08 [-0·18 to 0·02] *p* = 0·11 |
| **Data included in models** | | | | | |
| Participants | | 140 | 140 | 140 | 140 |
| Observations | | 420 | 420 | 420 | 420 |

Each model was estimated for 20 datasets for which missing data were replaced using multiple imputation by joint modelling; each model was then pooled to produce the parameters displayed above. Inspecting the table above, the parameter estimates and *p*-values do not change across Models 1–4; however, the intercept was different in each of the models (data not shown). CI = confidence interval. CMBAS = caring mindfulness-based approach for seniors. HSMP = health self-management programme. NfL = neurofilament light. V1 = baseline visit. V2 = post-intervention visit. V3 = follow-up visit.

**Table S5: Observational studies of longitudinal plasma NfL levels in older adults without objective cognitive impairment**

| **Study authors** | **Cohort name** | **Population type^a^ and sample size** | **Age at baseline, yrs (x̅ ± SD)** | **Average follow-up length (yrs)^b^** | **Baseline NfL, pg/ml (x̅ ± SD)** | **NfL mean change vs. baseline (scaled to 6 months; pg/ml)** | **NfL mean % change vs. baseline (scaled to 6 months)** |
| --- | --- | --- | --- | --- | --- | --- | --- |
| Baldacci et al.^1^ | INSIGHT-preAD study | SMC (*n* = 79)^c^ | 76·1 ± 3·5^d^ | 1·0^e^ | 29·8 ± 6·9 | +0·7 | +2·3% |
| Chatterjee et al.^2^ | AIBL | CU (*n* = 120)^f^ | 74·1 ± 6·3 | 1·5^g^ | 23·3 ± 11·3 | +1·0 | +4·5% |
| Hu et al^3^ | ADNI | CU (*n* = 243)^h^ | 73·0 ± 5·9 | NR^i^ | 33·8 ± 13·9 | +1·3 | +3·8% |
| Khalil et al ^4^ | ASPS-Fam | Stable CU (*n* = 95)^j^ | 64·0 ± 9·5 | 5·9^k^ | 31·7 ± 13·7 | NR^l^ | +2·3% |
| Verberk et al.^5^ | SCIENCe/ ADC | SCD (*n* = 92)^m^ | 61·0 ± 9·0^4^ | 2·6^n^ | 11·0 ± 6·0^4^ | +0·7 | +6·5% |

A number of longitudinal NfL studies were considered for this table, with only the most relevant included· Studies were omitted because data for CU participants were not reported separately from impaired participants,^6-8^ or because raw follow-up NfL values were not reported or unavailable.^9,10^ AD = Alzheimer’s disease. ADC = Amsterdam Dementia Cohort. ADNI = Alzheimer’s disease neuroimaging initiative. AIBL = The Australian Imaging, Biomarker & Lifestyle Flagship Study of Ageing. ASPS-Fam = Austrian Stroke Prevention Family Study. Aβ = Amyloid-beta. CSF = Cerebrospinal fluid. CU = Cognitively unimpaired. INSIGHT-preAD = Investigation of Alzheimer’s Predictors in subjective memory complainers. x̅ = Mean. MCI = Mild cognitive impairment. ml = millilitre. NfL = neurofilament light. NR = Not reported. PET = Positron emission tomography. pg = picogram. SCD = Subjective cognitive decline. SCIENCe = Subjective Cognitive Impairment Cohort. SD = Standard deviation. SMC = Subjective memory complaints.

Superscripts: ^a^Population type refers to baseline diagnosis/cognitive status; in some studies, small minorities of participants had progressed to MCI or dementia by the follow-up visit. ^b^This column shows the follow-up visit *that we used to calculate* *the six-month change in NfL in the table* (i.e., for comparison with the SCD-Well study), *not the total length* of follow-up available in the respective study; where multiple follow-ups were available in a given study, change was calculated based on the *earliest* follow-up, as NfL levels appear to increase non-linearly with time (i.e., the rate of change increases; see ^4^); furthermore, note that the ‘average’ follow-up was reported differently by different studies – some reported the mean, others the median, while some reported the intended inter-visit interval (e.g., 12 or 18 months). ^c^Total sample was *n* = 316, but only *n* = 79 had follow-up NfL data available. ^d^Data refer to the total (i.e., larger) study sample; data for the subset of individuals with follow-up (included in the table) were not reported separately. ^e^Article just reports that participants were followed up at 1 year. ^f^Total sample was *n* = 225, but this also included participants with MCI or AD; the data in the table refer only to the 120 CU individuals at baseline (81 were Aβ-PET- and 39 were Aβ-PET+ at baseline). ^g^Article just reports that participants were followed up at 18 months. ^h^The sample included 130 CU who were Aβ-, and 113 who were Aβ+ on PET/CSF. ^i^Article just reports that ‘More than 75 percent of participants had at least three years of follow-up’ (the NfL rate of change data presented above were synthesised/scaled from Table 1 in ^3^). ^j^Total sample was *n* = 335, but only *n* = 95 had follow-up NfL data available. ^k^Mean follow-up was 5·9 ± 1·0 years. ^l^For NfL, only percentage change data (not raw values at follow-up) were reported. ^m^Total sample was *n* = 300, but only *n* = 92 had follow-up NfL data available. ^n^Participants were followed up at a median of 2·6 years (range: 0·9–14·2 years).

**References**

1. Baldacci F, Lista S, Manca ML, et al. Age and sex impact plasma NFL and t-Tau trajectories in individuals with subjective memory complaints: a 3-year follow-up study. *Alzheimers Res Ther* 2020; **12**(1): 147.

2. Chatterjee P, Pedrini S, Ashton NJ, et al. Diagnostic and prognostic plasma biomarkers for preclinical Alzheimer's disease. *Alzheimers Dement* 2022; **18**(6): 1141-54.

3. Hu H, Chen KL, Ou YN, et al. Neurofilament light chain plasma concentration predicts neurodegeneration and clinical progression in nondemented elderly adults. *Aging (Albany NY)* 2019; **11**(17): 6904-14.

4. Khalil M, Pirpamer L, Hofer E, et al. Serum neurofilament light levels in normal aging and their association with morphologic brain changes. *Nat Commun* 2020; **11**(1): 812.

5. Verberk IMW, Laarhuis MB, van den Bosch KA, et al. Serum markers glial fibrillary acidic protein and neurofilament light for prognosis and monitoring in cognitively normal older people: a prospective memory clinic-based cohort study. *Lancet Healthy Longev* 2021; **2**(2): e87-e95.

6. Mielke MM, Syrjanen JA, Blennow K, et al. Plasma and CSF neurofilament light: Relation to longitudinal neuroimaging and cognitive measures. *Neurology* 2019; **93**(3): e252-e60.

7. Naude JP, Gill S, Hu S, et al. Plasma Neurofilament Light: A Marker of Neurodegeneration in Mild Behavioral Impairment. *J Alzheimers Dis* 2020; **76**(3): 1017-27.

8. Rauchmann BS, Schneider-Axmann T, Perneczky R. Associations of longitudinal plasma p-tau181 and NfL with tau-PET, Aβ-PET and cognition. *Journal of Neurology, Neurosurgery &amp;amp; Psychiatry* 2021; **92**(12): 1289.

9. Sugarman MA, Zetterberg H, Blennow K, et al. A longitudinal examination of plasma neurofilament light and total tau for the clinical detection and monitoring of Alzheimer's disease. *Neurobiol Aging* 2020; **94**: 60-70.

10. Sun Y, Tan L, Xu W, et al. Plasma Neurofilament Light and Longitudinal Progression of White Matter Hyperintensity in Elderly Persons Without Dementia. *J Alzheimers Dis* 2020; **75**(3): 729-37.
